# The Atlanta School of Medicine

**Published:** 1905-11

**Authors:** 


### THE ATLANTA SCHOOL OF MEDICINE.

THE Atlanta School of Medicine was organized and formally opened on October 3d.

At the opening exercises addresses were made by Drs. W. S. Kendrick, G. H. Noble, E. G. Jones and others, who outlined the policy of the new school and forecasted for it a prosperous career. The new school has secured temporary quarters in the Masons' Annuity Building, corner of Edgewood avenue and Ivy street, which it occupies jointly with the Atlanta Dental College. The trustees and faculty contemplate the erection in the near future of a commodious building, but have in the meantime thoroughly equipped their present quarters for medical teaching in its various branches.

The faculty is composed of the following gentlemen:

W. S. Kendrick, M.D., Professor of Medicine.

C. D. Hurt, M.D., Professor of Therapeutics.

Geo. H. Noble, M.D., Professor of Abdominal Surgery and Clynical Gynecology; Dean of the Faculty.

J. M. Crawford, M.D., Professor of Diseases of the Eye, Ear, Nose and Throat.

E. C. Davis, A.B., M.D., Professor of Gynecology and Obstetrics.

L. C. Fischer, M.D., Professor of Anatomy and Clinical Surgery.

Edward G. Jones, A.B., M.D., Professor of Gynecology and Obstetrics.

James L. Campbell, M.D., Professor of Surgical Anatomy and Clinical Surgery.

R. T. Dorsey Jr., B.S., M.D., Professor of Clinical Medicine.

F. K. Boland, A.B., M.D., Professor of Operative and Clinical Surgery and Demonstrator of Anatomy.

Hansell Crenshaw, M.D., Professor of Materia Medica and Chemistry.

Stewart R. Roberts, A.B., M.S., M.D., Professor of Physiology.

R. B. Ridley, Jr., M.D., Clinical Professor of Diseases of the Eye, Ear, Nose and Throat.

E. C. Thrash, M.D., Lecturer on and Demonstrator of Histology, Pathology and Bacteriology.

Thomas H. Hancock, M.D., Lecturer on Casualty Surgery and Clinical Professor of Surgery.

L. B. Clark, M.D., Lecturer on Diseases of Children.

O. T. White, M.D., Lecturer on Physical Diagnosis and Instructor in Gynecology.

W. B. Emery, B.S., M.D., Clinical Lecturer on Genito-Urinary Diseases.

E. G. Ballinger, M.D., Lecturer on Genito-Urinary Diseases.

W. E. Quillian, A.B., M.D., Lecturer on Dermatology.

W. B. Jones, A.B., M.D., Instructor in Abdominal Surgery and Clinical Gynecology.

J. Ross Simpson, M.D., Instructor in Anatomy.

A. P. Flowers, A.B., M.D., Instructor in Medica and Chemistry.

J. H. Crawford, M.D., Instructor in Diseases of the Eye, Ear, Nose and Throat.

John S. Hurt, A.B., M.D., Instructor in Therapeutics.

C. D. Heard, M.D., Instructor in Medicine.

C. O. Smith, M.D., Instructor in Clinical Medicine.

F. M. Sutton, M.D., Instructor in Obstetrics.

T. C. Longino, M.D., Assistant Demonstrator of Anatomy.

L. L. Moye, M.D., Assistant in Medicine and Gynecology.

Archibald Smith, M.D., Assistant in Obstetrics.

J. H. Kelly, M.D., Assistant in Clinical Surgery.

---

*Dr. Chas. Hicks*, of Dublin, will deliver special lectures on Medicine, and *Hon. Ruben Arnold* on Medical Jurisprudence.

